# Sound differences between electronic and acoustic stethoscopes

**DOI:** 10.1186/s12938-018-0540-2

**Published:** 2018-08-03

**Authors:** Lukasz J. Nowak, Karolina M. Nowak

**Affiliations:** 1 Institute of Fundamental Technological Research, Polish Academy of Sciences, Pawinskiego 5B, 02-106 Warsaw, Poland; 2 Centre of Postgraduate Medical Education, Ceglowska 80, 01-809 Warsaw, Poland

**Keywords:** Stethoscope, Auscultation, Electronic stethoscope, Acoustic diagnostics

## Abstract

**Background:**

The area of application of electronic stethoscopes in medical diagnostics covers the scope of usability of the acoustic stethoscopes, from which they have evolved and which they could potentially replace. However, the principle of operation of these two groups of diagnostic devices is substantially different. Thus, an important question arises, regarding the differences in parameters of the transmitted sound and their potential diagnostic consequences in clinical practice.

**Methods:**

In order to answer this question, heart auscultation signals are recorded using various stethoscopes and divided into short fragments based on the analysis of the synchronized recordings of electrocardiogram signals. Next, a dedicated algorithm is used to extract representative datasets for each case, which are then analyzed for their acoustic parameters. Four different electronic stethoscopes were investigated, together with an acoustic stethoscope as a reference point.

**Results:**

The determined acoustic characteristics of the considered stethoscopes differ significantly between each other.

**Conclusions:**

The differences in sound transmitted by various stethoscope models may translate into significant differences in quality of the obtained diagnosis. It is also pointed out, that the terminology and application guidelines regarding the electronic stethoscopes are misleading and should be changed.

## Background

Acoustic stethoscopes, invented over two centuries ago, are the most commonly used medical diagnostic devices and the symbol of healthcare professionals. They have simple, mechanical construction. A chestpiece picks up the sounds from the body of an auscultated patient, and a pair of hollow tubes transmits them to the ears of a physician. The construction and parameters of the individual parts of a stethoscope are supposed to have influence on the characteristics of the transmitted sound—its level and amplification or damping of various frequency components [1–7]. However, the results of some recently published investigations [8, 9] show, that this relation is non-trivial, and that the current state of art in the field is far from fully understanding. Especially, the role of the diaphragm of a chestpiece in filtering low-frequency signal components, as described in the literature, is questioned in the light of the new findings [8, 9].

The electronic stethoscopes are based on completely different operating principles. Their chestpiece is a contact microphone, which converts the vibrations of an underlying skin into an electric signal. Various models of electronic stethoscopes use different types of transducers, with different acoustic characteristics (see, for instance, [10, 11]). The electronic signal is amplified, filtered, and fed to a speaker or headphones.

Auscultation sounds transmitted through a stethoscope are quiet, and their dominant frequency components are localized near the lower limit of the audibility range. The diagnostic information contained in the signal may have subtle character and be hard to distinguish. Thus, concluding the appropriate diagnosis is a derivative of three main factors: good hearing and experience of a physician, and acoustic parameters of a stethoscope. These parameters determine how specific component of transmitted auscultation sound, linked to specific pathological state, will be presented on the background of other sound components and noise.

The electronic stethoscopes may amplify the quiet components of the auscultation sounds. However, if other sounds and noise, with higher frequency components—localized within better audibility region—will be amplified as well, obtaining correct diagnosis could be not possible. Thus, the manufacturers of the electronic stethoscopes implement various kinds of selectable digital filters, in order to remove the unwanted frequency components from the signal. The characteristics of these filters are set in a way to mimic the assumed characteristics of acoustic stethoscopes—the bell and the diaphragm type chestpieces. Such a solution is intended to provide the sound similar to what the physicians are used to, but ensuring higher volume levels and thus improving the diagnosis capabilities. However, due to the mentioned new findings indicating that the actual filtering properties of the diaphragm are different than supposed [8, 9], an important question arises, if and to what degree the sound provided by the electronic stethoscopes is similar to the sound transmitted via acoustic stethoscopes. Possible differences in this regard may have important clinical consequences—not only due to the mismatch with experience and expectations of physicians, but also due to the fact, that the doctor conducting auscultation might not be aware, that some specific, important components of sound were removed or damped in a selected filtering mode.

A method for objective evaluation of acoustic parameters of electronic and acoustic stethoscopes, described in details in [8], is used in the present study. The method takes into account the complex influence of mechanical coupling between the body of an auscultated patient and the chestpiece. Thus, the obtained data reflect the actual behavior of the considered diagnostic devices under the real conditions of patient examination. The comparison of the determined characteristics of four popular models of electronic stethoscopes and a representative model of acoustic stethoscope is presented. The analysis of the obtained results allows to answer the question, if and to what extent the electronic stethoscopes mimic the actual parameters of the acoustic stethoscopes?

## Methods

Acoustic parameters of four different, commercially available models of electronic stethoscopes, denoted here as ‘A’, ‘B’, ‘C’, and ‘D’, were investigated. The specific brands and names are not provided, as the purpose of the present study is to introduce an objective method for evaluation of acoustic parameters and to point out discrepancies that might influence the diagnosis, and not to suggest or compare any specific brands or devices. Each of the investigated stethoscopes is equipped with different type of electroacoustic transducer for converting vibrations of the underlying skin into an electric signal (‘A’—piezoceramic element, ‘B’—standard diaphragm with air-coupled electret microphone in the chestpiece, ‘C’—PVDF piezoelectric film, ‘D’—electrostatic capacitor-based contact microphone with pre-polarized electret diaphragm). Each of the stethoscopes has also a set of selectable digital filters. The parameters of these filters, accordingly to the data provided by the manufacturers, are presented in Table 1. As it can be seen, in all the cases the names of specific filter settings are referred to the bell and diaphragm chestpieces of an acoustic stethoscope, and constitute the use guidelines for a physician. What is interesting, is that different manufacturers provide different frequency ranges assigned to the corresponding operating modes.

**Table 1:** Description of the available operating modes—digital filter settings—for the investigated electronic stethoscopes, accordingly to the data provided by the manufacturers

Stethoscope ‘A’ (Hz)	Stethoscope ‘B’ (Hz)	Stethoscope ‘C’ (Hz)	Stethoscope ‘D’ (Hz)
Bell mode: 20–200	Bell mode: 20–200	Bell mode: 15–200	Bell mode: 60–500
Diaphragm mode: 100–500	Diaphragm mode: 200–500	Diaphragm mode: 100–500	Diaphragm mode: 80–500
Wide mode: 50–500	Wide mode: 20–1000	Wide mode: 15–4000	Wide mode: 20–2000

Accordingly to the assumed reference point for the parameters of the electronic stethoscopes, characteristics of an acoustic stethoscope equipped with double-sided chestpiece were also determined for comparison. A double chestpiece with bell and diaphragm allowed to directly compare the obtained results, with the parameters of auscultation sounds recorded using various electronic stethoscopes with different filter settings.

The auscultation sounds were recorded using an electret microphone placed in an earpiece, with the other earpiece sealed. Such a configuration allowed to take into account the influence of all the components of the acoustic pathway, and the obtained results accurately reflect the parameters of sound reaching the ear of a physician during auscultation. The microphone was connected to ZOOM H4N audio recorder. The only exception was the electronic stethoscope ‘D’, which, unlike all the other investigated devices, is not equipped with built-in speaker, traditional tubing and binaural with earpieces. Instead, it allows to connect any type of headphones with standard 3.5 mm jack plug. In this case, the audio recorder was connected directly to the headphone output of the stethoscope. Thus, one should keep in mind, that the results obtained in this case do not take into account the influence of the acoustic characteristics of headphones used for listening. This impact will be different for various models of headphones.

Heart auscultation sounds were selected as test signals for measurements of acoustic characteristics of the considered stethoscopes. This is justified by several important issues. Using auscultation sounds instead of external sound source, such as, for instance, a loudspeaker with signal generator is necessary in order to take into account the effects related to mechanical coupling of the stethoscope chestpiece with the body of an auscultated patient. This is the only way, in which the obtained results will reflect the actual characteristics of a stethoscope during an auscultation examination. In order to be able to compare the results obtained for various devices, a representative dataset for each case, selected from a large set of synchronously recorded acoustic events with parameters as close as possible, is required. The heart auscultation signals are the most suitable for such an analysis. It is also not without significance, that the heart auscultation is one of the most fundamental and most often performed patient examination carried out using a stethoscope, and that the differences in acoustic parameters of various chestpieces or filter settings are most often explained referring to the heart auscultation.

The bioacoustic signals were recorded during heart auscultation of a healthy male volunteer (aged 33, with BMI equal 24.8), at the mitral site. At the same time, the electrocardiography (ECG) signals from custom built electrocardiograph were synchronously recorded. Next, using the developed Matlab scripts, an automatic ECG signal analysis was performed. Based on the identified locations of the ECG waves, the acoustic signal was divided into short parts, defined as acoustic events. Each acoustic event was 0.557 s long and contained sound related to a single heartbeat. From the set of all the acoustic events obtained for each of the investigated stethoscopes, a subset of the acoustic events most similar to each other were selected. For this purpose, the value of normalized cross-correlation function was determined for each pair of acoustic events, and only the elements with values higher than a given threshold were selected for further analysis. The described procedure is described more in details in [8]. Such an approach allows to reject signals containing noise or other, incidentally recorded body sounds, which—if not removed—could significantly affect the obtained results and misled the analysis. The subsets of representative signals for each case were statistically analyzed for their frequency content. The obtained results reflect the acoustic characteristics of the investigated devices operating in various modes, under the conditions of the actual auscultation examination.

## Results

**Fig. 1:**
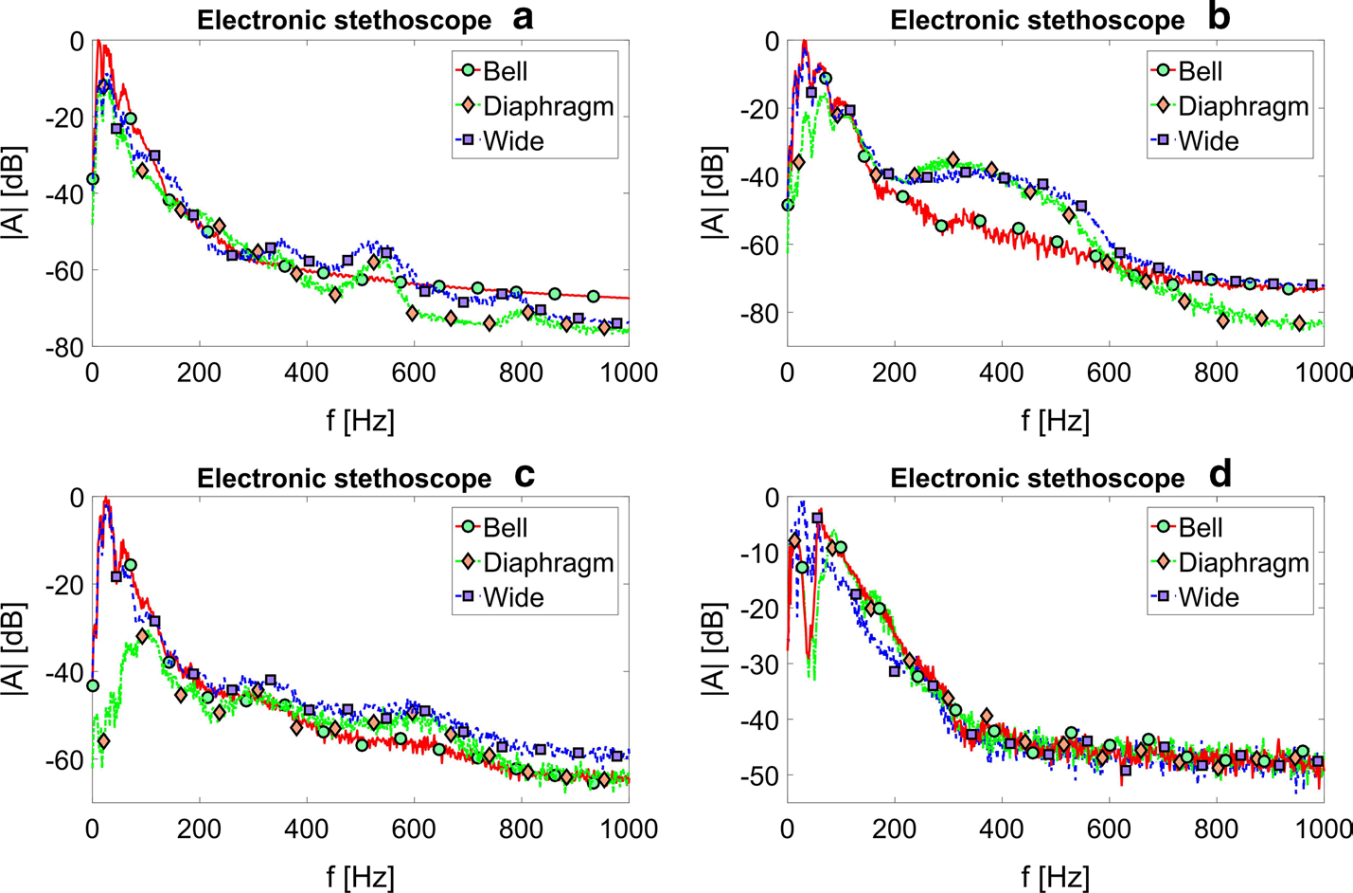
Electronic stethoscopes—acoustic spectra. The mean acoustic spectra of heart auscultation signals recorded using various investigated electronic stethoscopes: **a**–**d** operating in different available modes

Figure 1 presents the mean acoustic spectra of heart auscultation signals recorded using various investigated electronic stethoscopes, operating in different available modes. It is clearly visible, that different curves—representing different filter settings—vary significantly. Thus, the frequency content of the signal transmitted through the considered diagnostic devices was also different in each case.

**Fig. 2:**
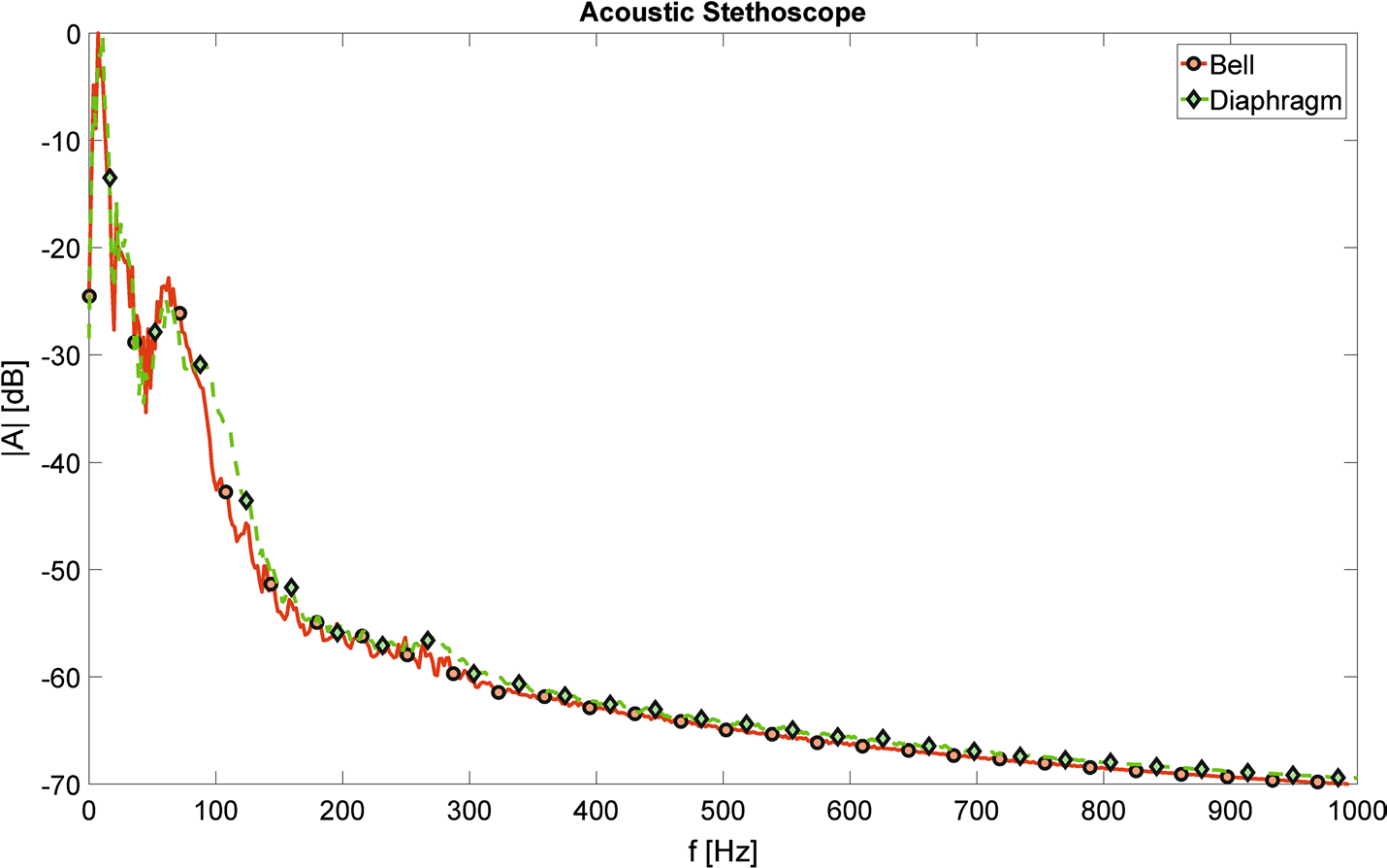
Acoustic stethoscope—acoustic spectra. The mean acoustic spectra of heart auscultation signals recorded using two different chestpieces of an acoustic stethoscope

Figure 2 presents analogous results obtained using the acoustic stethoscope, and two chestpieces: the bell and the diaphragm. It is clearly noticeable, that the differences between the curves in this case are much smaller than these observed in any of the investigated electronic stethoscopes.

**Fig. 3:**
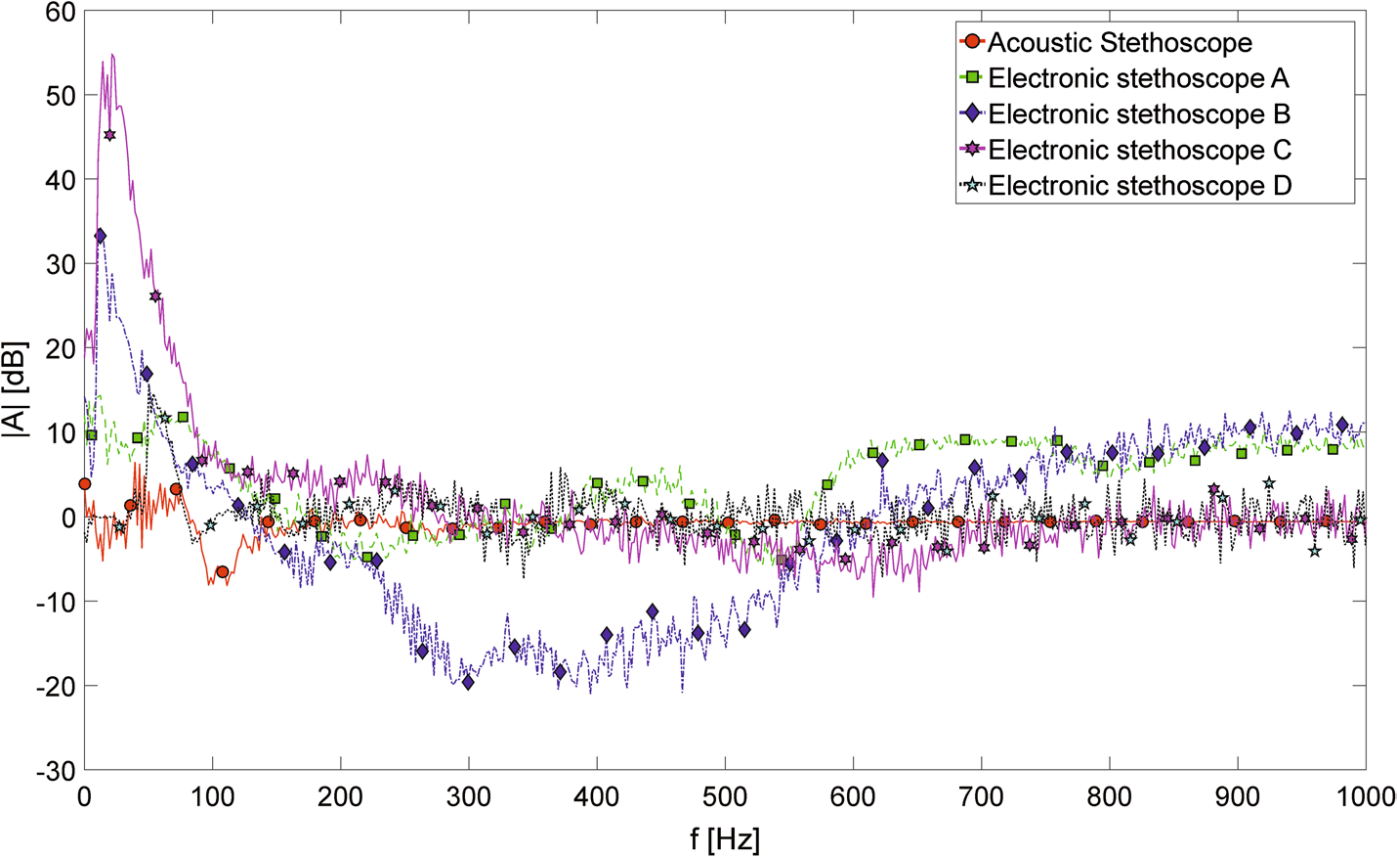
The computed values of bell-to-diaphragm coefficients. The computed values of bell-to-diaphragm coefficients—ratios of amplitudes of specific frequency components of signals recorded using various stethoscopes operating in bell and diaphragm modes, expressed in logarithmic scale

In order to better expose the difference in acoustic characteristics of the stethoscopes operating in bell and diaphragm modes, a bell-to-diaphragm coefficient, defined as:

1 $$\begin{aligned} A_{BD}\left( f\right) = 20 {\text{log}}_{10}\frac{ A_{B}\left( f\right) }{A_{D}\left( f\right) }, \end{aligned}$$

was computed for each of the investigated diagnostic devices. $A_{B}\left( f\right)$ denotes the amplitude of specific harmonic component at frequency *f*, obtained using the bell, while $A_{D}\left( f\right)$ denotes analogous value obtained using the diaphragm. The values of the determined coefficient $A_{BD}\left( f\right)$ for all the considered devices in the frequency range between 0 and 1000 Hz is presented in Fig. 3. The positive values indicate, that the amplitudes of specific harmonic components were grater in case of bell, and the negative values of the coefficient indicate, that the amplitude of a specific harmonic component was greater in the signal recorded using the diaphragm mode. As it can be seen, the presented $A_{BD}\left( f\right)$ values are in the range of about − 20 to 55 dB.

**Fig. 4:**
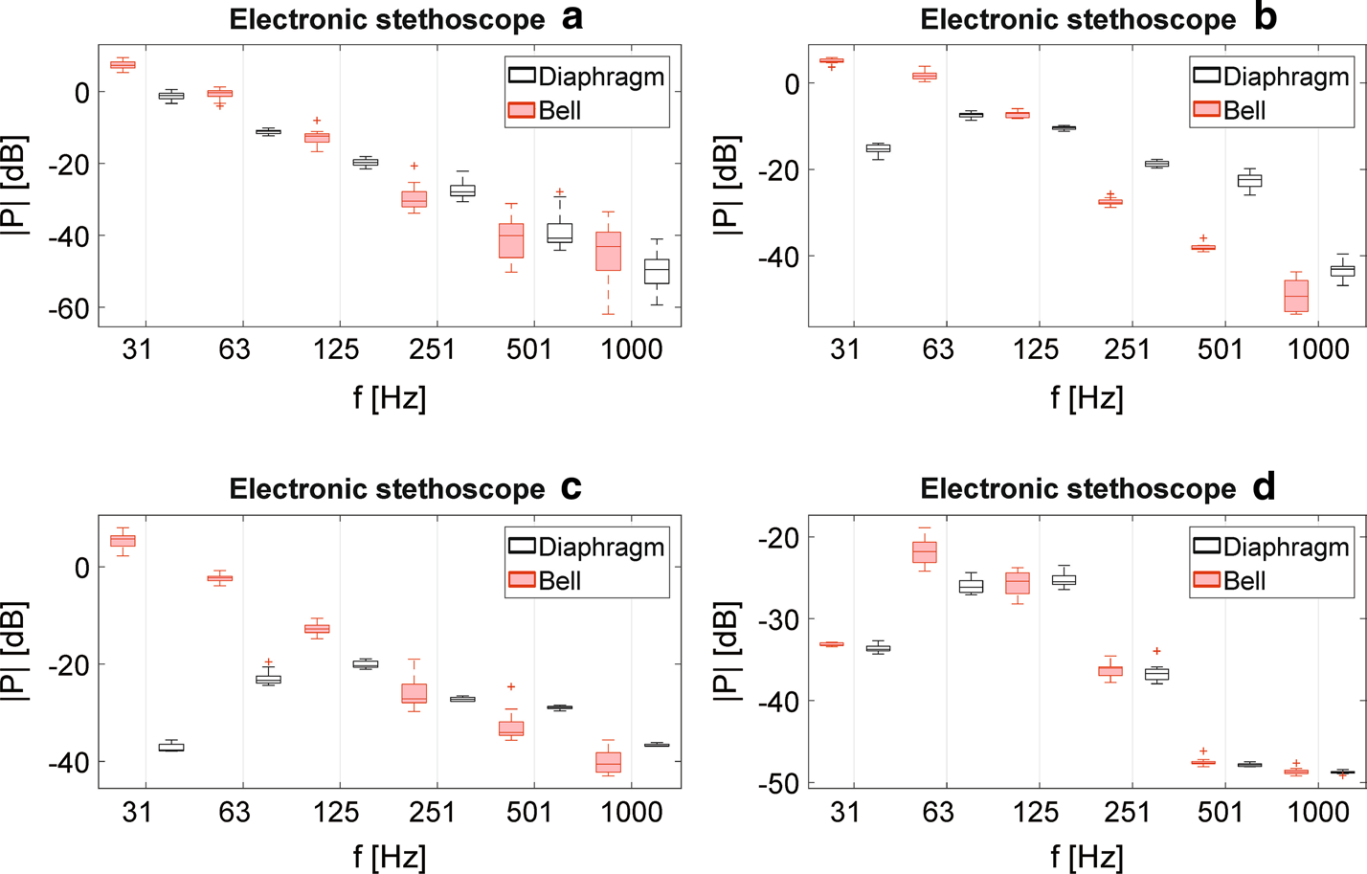
Electronic stethoscopes—octave analysis. Box plots presenting results of octave analysis of energy of signals recorded using various investigated electronic stethoscopes: **a**–**d** operating in different modes

**Fig. 5:**
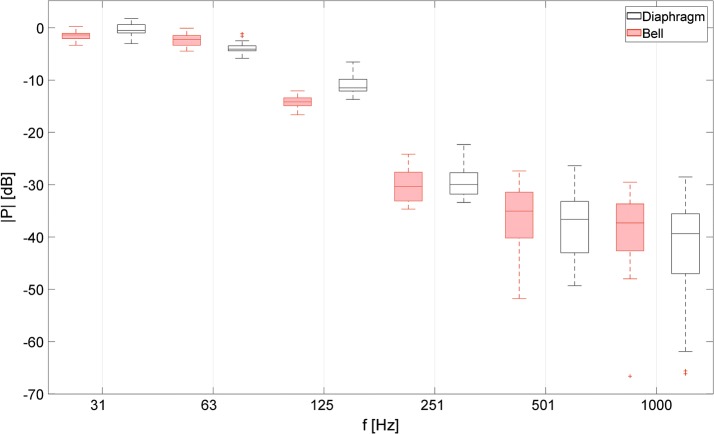
Acoustic stethoscope—octave analysis. Box plot presenting results of octave analysis of energy of signals recorded using two different chestpieces of an acoustic stethoscope

Figure 4 presents the results of the octave analysis of the auscultation signals recorded using various investigated electronic stethoscopes, operating in different available modes. The acoustic events extracted from the recordings were passed through an octave filter bank, and the signals at the outputs of each filters were statistically analyzed and presented in the box plots. The center line in each box indicates the median value, while the lower and upper edges represent the first and the third quartile, respectively. The whiskers of the boxes represent the data range, and the outliers (i.e. data with values beyond the maximum whisker length, set to 1.5 times the interquartile range) are marked with “+” signs. The analogous box plot presenting results obtained using the acoustic stethoscope is showed in Fig. 5.

## Discussion

The results presented in Figs. 1, 2, 3, 4, and 5 show the differences in frequency characteristics of heart auscultation sounds recorded using an acoustic stethoscope and different electronic stethoscopes, operating in various available modes. One can clearly see, that these characteristics differ significantly between all the considered cases. The amplitudes of spectral components were normalized with respect to the highest value in each case (i.e. to the highest amplitude for a given stethoscope and all the considered operating modes). The absolute values do not carry any relevant information in this case, as the gain of electronic stethoscopes can be adjusted in wide range. The volume was kept constant during measurements, making sure that the output signal is relatively loud but not over-driven.

A low-frequency character of the assumed test signals, with dominant harmonic components located below 200 Hz, is clearly visible in all the presented graphs. One may argue if signals, with such a limited spectrum, are appropriate or optimal for conducting the described measurements. However, one should also note, that only by using auscultation sounds it is possible to take into account effects related with mechanical coupling between the chestpiece of a stethoscope and the body of an auscultated patient. These effects play significant role in shaping the acoustic parameters of the considered devices, as both the diaphragm of an acoustic stethoscope and different kinds of electroacoustic transducers used in electronic stethoscopes change their mechanical behavior drastically in contact with a body. Thus, only results obtained in such a way refer to the actual patient examination. The heart auscultation signals are the only body sounds with subconscious and narrowly repetitive character, which can be recorded synchronously with high-quality electrocardiography signal for identification and extraction of specific acoustic events. The procedure described in the present study allows to obtain relevant results, directly related to the actual patient examination. It also allows to reject signals with noise and other, interfering random body sounds, by comparing and selecting only the most similar, representative subsets of acoustic events, thus significantly improving the quality of the obtained data. Finally, as the presented results clearly show, the parameters of the used test signals are more than sufficient to expose the differences in acoustic characteristics of the acoustic and electronic stethoscopes.

The determined characteristics of the electronic stethoscopes are in general quite consistent with the descriptions of the specific filter settings provided by the manufacturers in the datasheets and manuals, presented in Table 1. The characteristics of the implemented digital filters reflect the assumed behavior of the bell and diaphragm chestpieces of acoustic stethoscopes, as described in the literature (see, for instance, [1, 2]). The bandpass filters referred to as the bell mode, emphasize lower frequency components, while filters that are supposed to mimic the behavior of a diaphragm type chestpiece attenuate lower frequency components and emphasize higher frequency band. However, these assumed characteristics are inconsistent with the results obtained using an acoustic stethoscope.

Figures 2 and 5 present spectral characteristics of auscultation signals obtained using the acoustic stethoscope and two different chestpieces. Despite some slight differences in the lower frequency range, results obtained using the bell are very similar to the results obtained using the diaphragm. No supposed low- or high-pass filtering effects were observed. If that would be the case, significant discrepancies in the whole considered frequency band should be visible. The results presented as the boxplot in Fig. 5 confirm good consistency and low dispersion of the obtained data. These findings agree with conclusions presented in [8].

The spectral characteristics of auscultation sounds recorded using electronic stethoscopes, presented in Figs. 1 and 4, not only differ significantly from characteristics of the signals recorded using the acoustic stethoscope, but also between each other. It means, that the manufacturers of the electronic stethoscopes not only try to mimic non-existing properties of acoustic stethoscopes, but they are also doing it in their own, different ways. This can in obvious way lead to a confusion of a physician, who would have to use one of such devices, that he would not be used to.

The bell mode in electronic stethoscope ‘A’ removes much of the high-frequency content from the transmitted signal. It can be clearly seen on the corresponding graph in Fig. 1. The local maxima of the spectral components near 500 Hz and 800 Hz, clearly visible in the curves obtained using the diaphragm and wide modes are completely unnoticeable in the curve obtained using the bell mode. On the other hand, the bell mode emphasizes the frequency components below 100 Hz much more firmly, than the acoustic stethoscope. It is also worth noting, that the wide mode in the low frequency region is more similar to the diaphragm mode, than to the bell mode.

The differences between bell and diaphragm modes are even more visible in the spectral characteristics of the signals obtained using the electronic stethoscope ‘B’. The bell mode also emphasizes the lower frequency components and introduces relatively high attenuation in the frequency band above about 200 Hz. However, the wide mode in this case provides combination of the remaining two filter settings, and amplifying signal components in the whole considered frequency range. The largest differences between the spectral characteristics of signals obtained using bell and diaphragm-type filter settings are observed in the electronic stethoscope ‘C’. As it can be seen, in this case for the diaphragm mode the lowest frequency components are subjected to attenuation as high as almost 60 dB, with respect to the corresponding amplitudes of harmonic components of the signals recorded using the bell mode. Due to the dominant, low-frequency character of the considered heart auscultation signals, introducing such a filtering will result in significant worsening of audibility of these sounds. The cutoff frequency, for which the amplitudes of harmonic components are higher in the diaphragm mode, is equal about 500 Hz. It is also worth noticing, that the wideband mode in this stethoscope indeed provides highest amplification in the whole considered frequency range. It also becomes dominant over the diaphragm mode for frequencies greater than about 800 Hz.

As it can be seen from spectral curves obtained using the electronic stethoscope ‘D’ and presented on the corresponding graph in Fig. 1, the differences between different filter settings manifest themselves primarily in the low frequency band, below 300 Hz. In this region, many local minima and maxima, different between various modes, are observed. Due to the narrow-band character of the observed differences, they are not visible in the results of the octave-band signal power analysis, presented on the corresponding graph in Fig. 4. However, in general the frequency characteristics of the signals obtained using the bell- and diaphragm-alike filters are indeed most similar to each other among all the considered electronic stethoscopes, and thus the most similar to the actual characteristics of the acoustic stethoscope presented in Fig. 2.

The described differences between the acoustic characteristics of bell and diaphragm filtering modes (or, bell and diaphragm chestpieces) in the investigated stethoscopes are clearly visible on the graph presented in Fig. 3. The values of the introduced bell-to-diaphragm coefficient $A_{BD}\left( f\right)$ directly reflect the ratio of amplitudes of specific spectral components in logarithmic scale, for signals recorded using various devices and different operating modes. As it can be seen, for the acoustic stethoscope—which is assumed to be the reference point—these differences are relatively small, not exceeding 8 dB in the lowest frequency range, with many local minima and maxima. At frequencies above 200 Hz the values of the $A_{BD}\left( f\right)$ coefficient computed for the acoustic stethoscope are close to 0, which means, that the amplitudes of the corresponding components obtained using bell and diaphragm chestpiece are very close to each other. The curves computed for electronic stethoscopes clearly differ from the described characteristic. All of the investigated devices significantly emphasize lower frequency band, when operating in the bell modes—up to about 55 dB of difference in case of the electronic stethoscope ‘C’.

It is also important to emphasize, that the presented, measured acoustic characteristics of the electronic stethoscopes result not only from the chosen settings of implemented digital filters, but they are also influenced by the parameters of specific electroacoustic transducers and parameters of the whole (both electronic and acoustic) signal transmission path. This explains, why simple analysis based only on the parameters of the digital filters (if such, accurate enough, would be provided by the manufacturers) could not deliver relevant results from the clinical point of view, and justifies the use of much more complicated procedures taking into account the complex effects related to the mechanical coupling between the body of a patient and the chestpiece of a stethoscope, described herein. One should also notice, that the presented results concerning the electronic stethoscope ‘D’ do not take into account the additional influence of characteristics of the headphones on the resulting signal, as this device allows to connect any kind of headphones with standard 3.5 mm jack plug. In case of all other considered stethoscopes the presented results reflect directly the parameters of auscultation sounds heard by a physician.

It should be also noted, that unambiguous, direct comparison of acoustic characteristics of different brands and models of the electronic stethoscopes is impossible, due to the different possible volume settings. Using highest available gain levels often leads to signal clipping, making such results useless for the analysis. One of the possible solutions to this problem could be to define an arbitrary sound pressure level measure of the signal recorded using a microphone placed in an earpiece, and then trying to adjust the volume of each of the devices in order to obtain the same value of this measure. The recorded sounds, after segmentation and selection of the representative subsets of samples as described previously, could be used to compare frequency spectrum content or signal to noise ratios.

## Conclusions

The comparison between the obtained data clearly shows, that parameters of sound transmitted through various investigated models of electronic stethoscopes differ significantly both between each other and between the parameters of sound transmitted through the acoustic stethoscope. The names of different filtering modes implemented in electronic stethoscopes—which are in all cases referred to bell- and diaphragm-type chestpieces—instead of being guidelines for physicians are in fact misleading in terms of the actual acoustic parameters and expected sound characteristics.

A separate question remains how the described differences in objective acoustic parameters translate to subjective auditory sensations and, eventually, to the diagnosis drawn based on the auscultation examination. This is undoubtedly an important issue, as the acoustic stethoscope remains the most widespread medical diagnostic device, and the electronic stethoscopes are often regarded as its future successor. The results presented herein form a strong basis for further investigations in this direction, as they allow to refer any subjective data to the actual mechanisms and phenomena underlying auscultation.

The presented results do not specify whether any of the shown characteristics is worse or better than the others—they only show, that these characteristics differ significantly between each other. It seems very interesting why the manufacturers of different models of the modern electronic stethoscopes, instead of focusing on the search for optimal digital filters, that would allow to improve the diagnosis of various pathological states based on auscultation, try to mimic the non-existing behavior of the acoustic stethoscopes. Modern electronic signal processing techniques and devices create almost unlimited possibilities and great opportunities for further development of the stethoscopes and auscultation in general. However, in order to take advantage of these possibilities it might become necessary to break with previous routines and habits.
